# New Drugs on the Internet: The Case of Camfetamine

**DOI:** 10.1155/2014/419026

**Published:** 2014-07-16

**Authors:** Eduardo Cinosi, Ornella Corazza, Rita Santacroce, Matteo Lupi, Tiziano Acciavatti, Giovanni Martinotti, Massimo di Giannantonio

**Affiliations:** ^1^Neuroscience and Imaging Department, Chair of Psychiatry, “G. d'Annunzio” University, Via dei Vestini 131, 66100 Chieti, Italy; ^2^School of Life and Medical Sciences, University of Hertfordshire, College Lane, AL10 9AB Hatfield, UK

## Abstract

*Introduction.* The number of new psychoactive substances (NPS) advertised for sale online is constantly increasing and it has become a phenomenon of global concern. Among NPS, Camfetamine has been rediscovered as recreational drug in 2011. Very little information is still available in the scientific literature on its nature and potential health risks. *Methods.* Data in scientific literature were integrated with a multilingual qualitative assessment of a range of online resources over the period of 32 months (May 2011–January 2014). *Results. N*-Methyl-3-phenyl-norbornan-2-amine (Camfetamine) may act as an indirect dopaminergic agonist in the central nervous system and may have mild-moderate opioid activity too. There are no current epidemiological data about recreational use of Camfetamine; our research shows that it is indeed used especially by individuals with a history of recreational polydrug misuse. It facilitates mental alertness, induces relaxation, and, unlike many other stimulants, seems not to be associated with severe physical effects. Valid causes for concern issued in our research may be Camfetamine intravenous or intramuscular administration as well as its use in conjunction with other psychoactive substances. *Conclusions.* It is here highlighted that more large-scale studies need to be carried out to confirm and better describe both the extent of Camfetamine misuse and possible psychotropic/adverse effects.

## 1. Introduction

**Figure 1:**
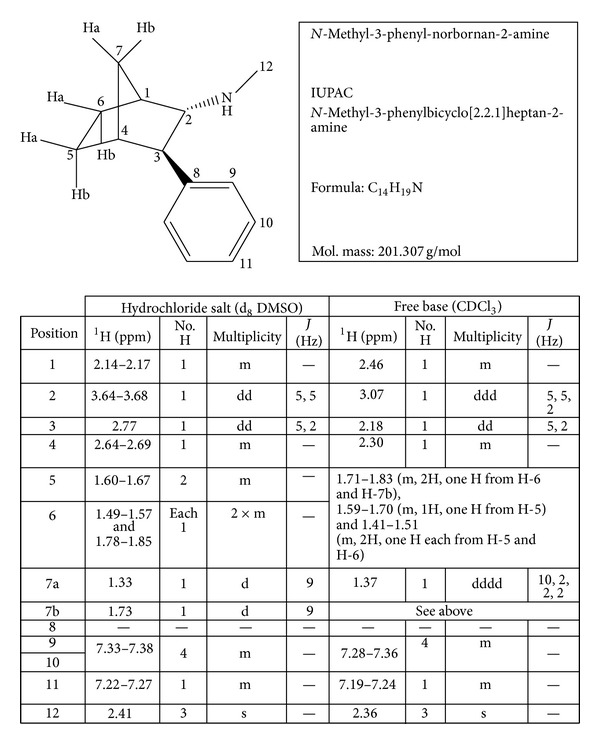
Chemical characterization of* N*-methyl-3-phenyl-norbornan-2-amine, modified from [10].

**Figure 2:**
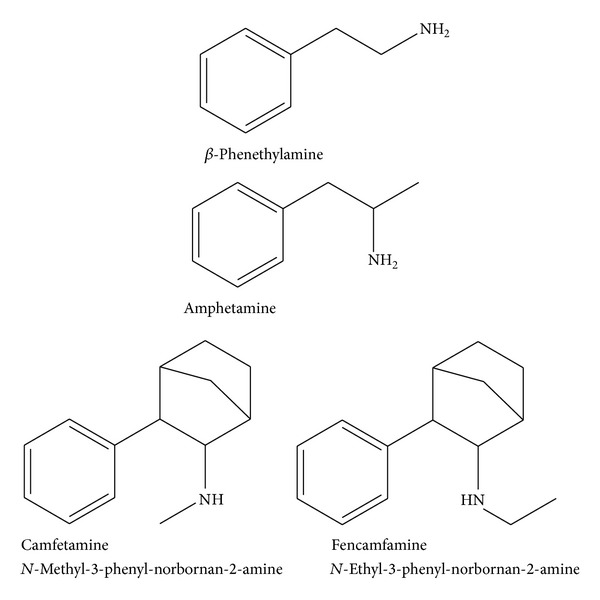
Camfetamine and its* N*-ethyl analogue Fencamfamine are heterocyclic amphetamine derivatives. Modifying the aminoalkyl side chain into heterocyclic structures still preserves the basic *β*-phenethylamine structure and retains the central stimulant activity without marked anorexigenic and cardiovascular side effects.

The term “new psychoactive substances” (NPS) had been legally defined earlier by the European Union as a new narcotic or psychotropic drug, in pure form or in a preparation, that is, not scheduled under the Single Convention on Narcotic Drugs of 1961 or the Convention on Psychotropic Substances of 1971, but which may pose a public health threat comparable to that posed by substances listed in those conventions (Council of the European Union decision 2005/387/JHA) [1]. That legal definition is now widely used and has also been adopted by the European Monitoring Centre for Drugs and Drug Addiction (EMCDDA) [2]. The term “new” does not necessarily refer to newly synthetized substances but to a wide range of products that have recently become available on illicit drug market. Many NPS were indeed synthesized and patented decades ago for research purposes, but only recently their chemistry or process of synthesis has been rediscovered or slightly modified to produce effects similar to known illicit substances [3]. In this paper, the authors focus on this specific compound* N*-methyl-3-phenyl-norbornan-2-amine (Camfetamine), a stimulant drug with effects similar to amphetamine. Precisely,* N*-methyl-3-phenyl-norbornan-2-amine was developed and patented as an analeptic by Merck, Darmsradt, in 1961 (Figure 1). Its synthesis was also described by a group in Smith Kline and French Laboratories when it was prepared as a part of a study to elucidate the stereochemistry of 3-phenyl-norbornan-2-amine [4]; however, it was never commercialized. Indeed, the* N*-ethyl analogue of 3-phenyl-norbornan-2-amine (Fencamfamine) is better known and has been sold under the trade name Reactivan as a central nervous system (CNS) stimulant and appetite suppressant, also prescribed for reduced performance and for rehabilitation after prolonged and debilitating diseases [5] (Figure 2). Fencamfamine seems also to have been frequently reported as dope substance in sport [6]. While Fencamfamine is listed in the Schedule IV of the UN Convention on Psychotropic Substances, Camfetamine is not listed and appears worldwide mostly unregulated; some exceptions are represented by Portugal, Hungary, and Poland [7–9]. Recently, the Association of Independent Research Chemical Retailers (AIRCR), an umbrella organization for a number of online vendors, has redeveloped it for use as a recreational drug [10]. According to our online monitoring activity, the first reports about Camfetamine misuse appeared in May 2011 [11]. Concomitantly, in 2011 Camfetamine was reported among NPS seizures by official authorities in several countries as United Kingdom, Finland, and Israel [3, 7, 8]. In 2013, Camfetamine was identified in mixtures with methoxetamine, caffeine, taurine, and methiopropamine in Germany [12]. However, very little information is still available in the scientific literature on Camfetamine nature and potential health risks related to its use as a recreational drug.

## 2. Materials and Methods 

The literature on Camfetamine was searched in three databases: PsycINFO, PubMed, and Medscape. Keywords used to carry out the database searches included the following: “*N*-methyl-3-phenyl-norbornan-2-amine,” “Camfetamine,” and “*N*-methyl-3-phenylbicyclo[2.2.1]heptan-2-amine.”

Considering the limitation of peer-reviewed data in scientific literature, results were integrated with a multilingual qualitative assessment of a range of websites, drug fora, and other online resources (i.e., e-newsgroups, chat-rooms, mailing lists, e-newsletters, and bulletin boards). This was carried out using the Google search engine in two languages (English and Italian). The online assessment was carried out over the period of 32 months (May 2011–January 2014) and involved the close monitoring of the sources listed above. Once the Camfetamine availability of information was identified on these websites, further specific searches were carried out for narratives focusing on the following issues: (i) the nature of its effects on users, including adverse reactions; (ii) motivations behind its recreational use and possible trends of misuse, with particular attention to polydrug misuse/idiosyncratic combinations; (iii) any other relevant information. For the purpose of reporting the results in this paper, any data collected from online fora, such as usernames and complete URLs for specific threads that were considered personal identifiable, were anonymized. Permission for the study was granted by the School of Pharmacy Ethics Committee, University of Hertfordshire, Hatfield, UK (November 2013; PHAEC/10-42).

## 3. Results and Discussion

### 3.1. Pharmacology and Possible Mechanisms of Action of Camfetamine

When considering the myriad structures of synthetic amphetamines and MDMA derivatives it is essential to return to the structural backbone that is common amongst them—the *β*-phenylethylamine molecule. Phenylethylamine (from the Greek root* Phainein*, meaning to show or to illuminate) is the term used to describe any structure derived from an aromatic group adjoined to a terminal amine by an ethyl group (Figure 2). This apparent structural simplicity belies the vast number of novel psychoactive substances and their corresponding and varied psychoactive effects that can be produced from modifications to the phenylethylamine backbone [13]. Camfetamine (*N*-methyl-3-phenylnorbornan-2-amine) and its* N*-ethyl analogue Fencamfamine are heterocyclic amphetamine derivatives (Figure 2). Some authors suggest that modifying the aminoalkyl side chain into heterocyclic structures still preserves the basic *β*-phenethylamine structure and retains the central stimulant activity without marked anorexigenic and cardiovascular side effects [14].

Only two results are currently displayed in scientific literature on Camfetamine. In the first paper, Kavanagh and colleagues [10] have described the synthesis of* N*-methyl-3-phenyl-norbornan-2-amine (chemical name* N*-methyl-3-phenylbicyclo[2.2.1]heptan-2-amine, Camfetamine shown in Figure 1), its characterization and interpretations of its electron impact, and electrospray ionization mass spectra. The authors highlight that the appereance of a new recreational drug always poses the problem of obtaining an authentic reference standard for use in forensic analysis [10]. In the second paper, Welter and colleagues [15] have aimed to study the metabolic fate and the detectability of Camfetamine (CAM) in rat urine and to elucidate which cytochrome-P450 (CYP) isoenzymes are involved in the main metabolic steps. The following main metabolic pathways were deduced: N-demethylation, aromatic mono or bis-hydroxylation followed by methylation of one hydroxy group, hydroxylation of the norbornane ring, combination of these steps, and glucuronidation and/or sulfation of the hydroxy metabolites [15]. The progressive metabolization was catalyzed by CYP2B6, CYP2C19, CYP2D6, CYP3A4, and CYP1A2 [15]. The authors showed that the intake of a common user's dose of CAM could be confirmed in rat urine, with the hydroxy-aryl CAM and the corresponding glucuronide metabolites being the most abundant [15].

Thus, nowadays very little is known about the pharmacology of Camfetamine but it may be expected to have similar properties to its* N*-ethyl analogue Fencamfamine (FCF) [10]. However, Camfetamine is potentially less lipophilic (calculated log* D* (pH 7.4) = 0,01 whereas the value Fencamfamine is 0,31) and this may result in lower bioavailability across the blood brain barrier [10]. Fencamfamine (FCF) is a psychostimulant that has complex effects in the central nervous system. Regarding acute effects, some studies suggest that FCF releases dopamine (DA) from both *α*-methyl-p-tyrosine and reserpine pools at dopaminergic cerebral nerve terminals [16]. FCF increased the homovanillic acid (HVA) levels in nucleus accumbens, tuberculum olfactorium, and corpus striatum and the dihydroxyphenylacetic acid (DOPAC) levels in the nucleus accumbens; on the other hand, amphetamine (AM) decreased the DOPAC levels in the 3 structures [16]. These data indicate that FCF and AM may differ from each other on particular mechanisms by which catecholamines are released [17].* In vitro*, FCF blocked [^3^H] catecholamine uptake into brain synaptosomes with potency similar to AM, but the [^3^H] dopamine releasing activity in striatal slices was low compared to AM [17]. Thus, FCF seems to produce its effects by predominant inhibition of DA reuptake, while AM seems to act as a DA releaser [18]. These results were confirmed by microiontophoretic approach [19]. FCF showed low affinity for binding sites of DA receptors ligands and little effect on wall brain monoamineoxidase activity [17, 20]. Considering all the evidences, we support the view that CAM, as FCF, may act as an indirect dopaminergic agonist in the central nervous system [21]. Moreover, interesting findings suggest that uptake inhibition and the release properties of FCF may undergo daily variation; the circadian time-dependent effects of FCF might be related to a higher susceptibility of dopamine presynaptic terminals to the action of FCF during the light phase which corresponds to the rats' resting period [22].

Regarding long-term effects, it is well known that repeated use of CNS stimulants can lead to either tolerance or sensitization [23, 24]. Preclinical studies showed that the behavioral effects of FCF include activation of locomotor, an increase in the stereotyped sniffing, and paradoxical decrease in rearing and behavior. At high doses, it induces stereotyped behavior [16]. It was also shown that FCF can act as a positive reinforcer and was hypothesized that opioid receptors mediate the reinforcing effects of FCF [25]. Furthermore, changes in the sensitivity of pre- or postsynaptic DA receptors might underlie both tolerance and sensitization to the effects induced by long-term FCF administration [26, 27]. Some authors suggest that chronic FCF treatment is linked to a modification in Na,K-ATPase activity through the cyclic AMP-dependent protein kinase, nitric oxide synthase (NOS) activity, and cyclic GMP levels in the nucleus accumbens (NAc) and striatum (ST) [28].

Clinical data on Fencamfamine administration in healthy volunteers showed that its stimulant effects are more pronounced with higher dose (50 mg), its paradoxically sedative effects are obtained with lower dose (25 mg), and it impairs cognitive functioning, increases awakeness, and depresses REM sleep [29]. To date, there is no human data that can determine the chronic toxicity, the dependence, and abuse liability of Camfetamine.

### 3.2. Information on Camfetamine Availability and Consumption

In general, the Internet seems to be an important source to obtain NPS worldwide. The significant informational, promotional, and distributional capacity of the Internet plays an important role in the NPS market and global web-based marketing and distribution distinct from illegal street markets has developed in past years [30]. Our research has identified 12 different websites in which customers can easily buy Camfetamine online [31–42]. The Internet seems to offer many advantages to NPS suppliers as it provides access to a vast number of potential users and suppliers who do not need large upfront investments and can retain some level of anonymity. It is important to point out that, in many cases, sellers fail to list ingredients hence raising further concerns in terms of the presence of contaminating agents, side effects, or drug interactions of the advertised product [43, 44]. According to our searches, the first online reports on Camfetamine use as recreational drug appeared in May 2011 both in Italy and UK with enthusiastic expectations among users and potential users [45, 46]. Camfetamine is sold as a white or brownish-yellow, clumpy, odourless, and salty powder. The average prices are €11: 250 mg; €20: 500 mg; €38 per gram; €70: 2 g; €160: 5 g. It is most commonly sniffed or taken by oral ingestion (frequent is the “bombing” technique, e.g., wrapping Camfetamine in a cigarette paper and swallowing it, or to melt it in drinks). Other ways of consumption include smoking, intramuscular injection, intravenous, and even rectal administration. Average doses range between 50 and 200 mg. Furthermore, redosing appears to be a common practice and typically involves the intake of more than one dose of 50–100 mg, with the total consumption up to 150–250 mg [11, 46–48].

*Re: Camfetamine. *An example of online report experience with Camfetamine available online at http://www.psychonaut.com/sintetici/38797-camfetamine.html (accessed on 11/1/2014).

40 Year old male, 20+ years of experience with most types of illegal drugs, and a dozen or so different RC's.

I received 500 mg of the re-crystallized product from my favourite, excellent vendor. It looks off-white with chunks and clumps, no smell that I noticed, though I didin't take it nasally as it looked like it would be a painful experience.

+0 h: 80 mg in a gelcap on an empty stomach, at the end of a hard day at work shovelling concrete all day in the sun, welp.
+1 h: Feeling slight stimulation, quite similar to Modafinil, but with even less of a hard edge. Modafinil only affects me at 400 mg doses, but at that level it can be slightly jittery, Camfetamine, so far, is more pleasant.
+2 h: Slightly more alert, mind seems to be working faster, I catch up on a couple of zeropunctuation videos and he sounds like he's speaking slower than usual.
+3 h: No euphoria, but I do feel good, especially considering the day I've had. I feel a bit hungry, but no real desire to eat, though probably could if I wanted.
+3.5 h: Plateaux I think, so I take another 50 mg. The reported feeling of “smoothness” is definitely a good description. No jitteryness, no anxiety like I often get. Blood pressure seems slightly raised, and occasionally think I may have the beginnings of a headache, but it never manifests. Pulse seems normal.
+4 h: Eat some food as my stomach is gurgling loudly. No problems eating, rather nice actually.
+5 h: Probably the most “high” so far, wish I was going out to a bar or the like, but just browse like a demon instead. I watch some iPlayer, and it also seems slow, this stuff might be a good study aid, or functional stim. Definitely recreational too though imho.
+6 h: It's 2 AM and normally Id be asleep after such a hard (week) day at work, but I'm still wide awake and feeling great.
+7 h: Been yawning for a bit now, though still very alert. I have shit to do tomorrow so take 21 mg Etizolam, go to bed and sleep for 7 hours.
+13 h: Feel fine, a bit groggy from the benzo perhaps, and slightly drained, perhaps because I would normal sleep for 9 hours.

*Summary. *Nice stim, smooth and clean, a calming effect too, as others have described. Not one for the true speed freaks, but I enjoyed it and will try again with a 120 ish dose.

I was very dubious about the reported opioid effect, but there may be some truth. I'm dpendant on dihydrocodeine (again, ffs) I take 480 mg in the morning and the same in the evening. Last night after taking the camfetamine, I had no desire for my DHC, felt none of the usual mild wd symptoms either.

Not totally convinced, but it seems possible it does have an opioid action. To know for sure, I'd have to take it, with no opiates for 2-3 days. Heh, that's not going to happen.

Not a great TR, the whole time I was on it I was trying to think of good ways to describe how I feel, or something to compare it to, but it is a weird one, not in a bad way, just not quite like anything else I've tried.

I can't imagine ever taking “real” speed again, too old, cant be arsed with staying awake for days. Or psychosis. This feels infinitely less toxic than the speed I've taken in the past, and is a quite contradictory substance, mellow speed? Smooth, calming, stimulation?

### 3.3. Desidered Effects and Side Effects

“Smooth” onset has been frequently reported (see Re: Camfetamine). The effects are described to reach the “high” within an hour and last for 4-5 hours [45, 46, 48, 49]. Unlike other stimulants, many users have reported the need to repeatedly redose this compound in order to get any appreciable stimulant or recreational value [46, 48, 50]. Desired effects include a marked improvement in mental alertness, feeling of clarity, and a stimulant effect followed by a sense of calmness and relaxation [45, 46, 48–50] (see Re: Camfetamine). Some experiences report that it also acts as a modest appetite suppressant and improves fatigue. Users report mood changes ranging from no mood enhancement to general pleasant mood or light euphoria at higher doses [45, 46, 48, 50]. On the other hand, users searching for strong euphoric/stimulant effects appear to be critical and disappointed about Camfetamine reporting that it is relatively ineffective and suggesting to choose other stimulants or to use it in combination with other substances [45–52].

Side effects manifested up to 24 hours after the intake including anxiety, headache, depressed or disphoric mood, unpleasant body sensations, and severe sleep impairment [45, 46, 48]. However, some users do not report any side effects. About adverse reactions, unlike “classic” stimulants such as cocaine or amphetamine, Camfetamine seems not to be commonly associated with severe physical sympathomimetic effects such as hypertension or respiratory difficulties [11]. Camfetamine is described by most of the users to moderately increase heart/pulse rate, give a light temperature increase, cause slight urinary retention, and dilate pupils [46, 48]. All users report, after insufflation, extremely unpleasant caustic burning sensations to nasal mucous membrane tissue associated with runny nose, squeezing, lacrimation, and corrosive feelings to the throat [45, 46, 48, 50]. Higher doses do seem to increase the stimulant effects of the drug but they also determine an increase in the toxicity. Worrisome data issued in our research are related to intramuscular or intravenous use of Camfetamine that appear to be particularly toxic. It can cause severe local pain in the site of puncture with marked vasoconstriction, painful muscle tension and stiffness, ataxia, blurred vision, muscle weakness, violent incontrollable diffuse tremors, bruxism, and dystonia. For this reason, some users do not recommend IV or IM administration even after complex chemical processes of purification and recrystallization [46, 48]. Within the sample considered in our online monitoring, Camfetamine is commonly taken in conjunction with many other psychoactive substances such as alcohol, cannabis, cocaine, heroin, amphetamine, Metamphetamine, MDMA as well as the less common Methiopropamine (MPA), 5,6-methylenedioxy-2-aminoindane (MDAI), methoxetamine (MXE), N,N-dimethyltryptamine (DMT), *α*-pyrrolidinopropiophenone (*α*-PPP), 6-(2-aminopropyl)benzofuran (6-APB), Kanna, Kratom, Dimethocaine, and Prolintane [45, 46, 48, 50]. This polydrug use might be associated with a wide number of unknown side effects/adverse reactions that are potentially lethal. Like for other stimulants, Camfetamine is also used in combination with various medications to self-treat unpleasant effects due to its intake: sedative/hypnotics for agitation and insomnia (Etizolam, Lorazepam, Temazepam, and Clomethiazole), anticonvulsant/myorelaxants for muscle tension and tremors (Pregabalin, Lamotrigine, and Tizanidines), and antiemetic for nausea (Domperidone) [45, 46, 48, 50]. Furthermore, many Camfetamine users admit to be under current psychiatric treatment (e.g., with antidepressants, mood stabilizers), without specifying the diagnosis, or under current opioid substitution/analgesic therapy (Methadone, Buprenorphine, Naltrexone, Tramadol, O-Desmethyltramadol, Codeine, Oxycodone, and Fentanyl) and state to mix and misuse also other products (e.g., with Methylphenidate and Modafinil) for recreational purposes [45, 46, 48, 50].

## 4. Conclusions

The amphetamine-type stimulants class has always been characterized by a large variety of substances. Among these, Camfetamine represents just one of the latest trends within the drug market. Starting from May 2011,* N*-methyl-3-phenyl-norbornan-2-amine has been redeveloped for use as a recreational drug. To the best of our knowledge, this is the first paper providing both an overview of the current scientific data available on Camfetamine and a critical analysis of the information related to its psychoactive effects, side effects, and use in combination with other drugs.

Camfetamine may act as an indirect dopaminergic agonist in the central nervous system and may have mild-moderate opioid activity too. It produces increased mental alertness, relaxation and, unlike many other stimulants, seems not to be associated with severe physical effects. Only little is known in terms of risks; one could argue about the possible risks associated with ingesting a drug that presents with potential for dependence and the anecdotal report on injecting use. A valid cause for concern issued in our research may be its use in conjunction with other psychoactive substances.

Nowadays, Camfetamine is largely available online and thus “just a click” away from our homes and potentially available to everyone. Moreover, the Internet serves as a repository of information for several groups of people and drug users can obtain information through online forums, chat rooms, and blogs and find out about new products. They can also communicate with other users on their experiences, the effects of the substances, and the recommended sources and avenues of delivery [53] (see Re: Camfetamine). The apparent possibility to purchase Camfetamine from Websites makes this drug very easily available to vulnerable individuals, including children and adolescents [54]. Vulnerable individuals might be encouraged by a range of widely available online comments/messages/videos related to Camfetamine intake experiences. This may be an issue of concern if one considers that an estimated 61% of young European people aged between 15 and 24 years typically quote the Internet as a potential source of information on drugs [55]. Furthermore, Camfetamine seems to be mostly unregulated and this may facilitate its popularity as well as the users' perception of risks associated with its consumption. The idea that legality can equate with safety still remains well grounded amongst some recreational users [56–60]. There are no current epidemiological data about Camfetemine use as recreational drug: our research shows that it exsists and mostly in individuals with a history of recreational polydrug misuse. Moreover, the fact that our research was carried out using the Google search engine just in two languages (English and Italian) might underestimate the Camfetamine diffusion.

A possible limitation of our analysis could be given by the fact that publicly available websites, fora, and similar sources were monitored. One could wonder about many limitations of carrying out a risk of misuse assessment of a drug while taking into account the online comments. First, it may be inappropriate to trust information obtained from the Internet without independent verification. Second, the present findings do rely on what is reported by users and we did not have any possibility here to ascertain if the substance the online alleged drug users were taking was indeed* N*-methyl-3-phenyl-norbornan-2-amine. Third, Camfetamine effects/adverse reactions are described in our qualitative analysis by a population of polyabusers as having most probably a high tolerance to many substances, and some of them declare even to be drug addicted or under current psychiatric treatment. Globally, Camfetamine users considered in our analysis, added together, are also intaking alcohol, cannabis, cocaine, heroin, amphetamine and synthetic amphetamine derivatives, Piperazine-based derivatives, Mephedrone, Pipradrol and derivatives, aminoindane analogues, Ketamine and derivatives, synthetic cannabinoid receptor agonists, Tryptamines, Benzofurans and Benzodifurans, natural product (Fungal and Herbal) novel psychoactive substances, Benzodiazepines, Barbiturates, anticonvulsant, antidepressants, and opioid substitution/analgesic therapies. Such a phenomenon constitutes a serious public health challenge: pharmacological, toxicological, and psychopathological effects due to interactions among all these substances may be unpredictable and fatal in vulnerable individuals. Moreover, such a chronic polydrug intake may lead to neurobiochemical CNS alteration that might make these polyabusers extremely difficult to be pharmacologically treated even by expert mental health professionals. On the other hand, online reports about the experience with Camfetamine seem genuine and many users illustrate their detailed experiences with Camfetamine as proper experiments (see Re: Camfetamine). Thus, in the absence of relevant peer-reviewed data, the online monitoring seems to be indeed the only method to obtain preliminary information about new and emergent phenomena. One could conclude that a constant web-monitoring activity with respect to drug-related issues is necessary to better understand the level of the diffusion of novel psychoactive substances such as Camfetamine. It is here suggested that better international collaboration levels may be needed to tackle the novel and fast growing phenomenon of novel psychoactive drugs availability from the web. Furthermore, it is here highlighted that more large-scale studies need to be carried out to confirm and better describe the extent as well as the risks of Camfetamine use in the European Union and elsewhere. Again, health and other professionals should be rapidly informed about this and other new and alerting trends of misuse. In this context, we suggest that the use of technological tools could be successfully incorporated in specific prevention programmes targeted at both health professionals and young people looking for reliable information about novel psychoactive substances.
